# A Multivariate Phenotypical Approach of Sepsis and Septic Shock—A Comprehensive Narrative Literature Review

**DOI:** 10.3390/medicina60111740

**Published:** 2024-10-23

**Authors:** Alina Tita, Sebastian Isac, Teodora Isac, Cristina Martac, Geani-Danut Teodorescu, Lavinia Jipa, Cristian Cobilinschi, Bogdan Pavel, Maria Daniela Tanasescu, Liliana Elena Mirea, Gabriela Droc

**Affiliations:** 1 Department of Anesthesiology and Intensive Care I, Fundeni Clinical Institute, 022328 Bucharest, Romania; alina_tita@yahoo.com (A.T.); cristina.martac@drd.umfcd.ro (C.M.); geani-danut.teodorescu0721@stud.umfcd.ro (G.-D.T.); laviniajipa11@yahoo.com (L.J.); 2 Department of Anesthesiology and Intensive Care I, Faculty of Medicine, Carol Davila University of Medicine and Pharmacy, 020021 Bucharest, Romania; 3 Department of Internal Medicine II, Faculty of Medicine, Carol Davila University of Medicine and Pharmcy, 020021 Bucharest, Romania; teodora.isac@umfcd.ro; 4 Department of Anesthesiology and Intensive Care II, Faculty of Medicine, Carol Davila University of Medicine and Pharmcy, 020021 Bucharest, Romania; cristian.cobilinschi@umfcd.ro (C.C.); liliana.mirea@umfcd.ro (L.E.M.); 5 Department of Anesthesiology and Intensive Care, Clinical Emergency Hospital, 010024 Bucharest, Romania; 6 Department of Physiology, Faculty of Medicine, Carol Davila University of Medicine and Pharmcy, 020021 Bucharest, Romania; bogdan.pavel@umfcd.ro; 7 Department of Medical Semiology, Faculty of Medicine, Carol Davila University of Medicine and Pharmcy, 020021 Bucharest, Romania; maria.tanasescu@umfcd.ro; 8 Department of Internal Medicine I and Nephrology, Faculty of Medicine, Carol Davila University of Medicine and Pharmcy, 020021 Bucharest, Romania

**Keywords:** sepsis, septic shock, omics, phenotype, precision medicine, multi-organ dysfunction, personalized therapy

## Abstract

Despite medical advances, sepsis and septic shock remain some of the leading causes of mortality worldwide, with a high inter-individual variability in prognosis, clinical manifestations and response to treatment. Evidence suggests that pulmonary sepsis is one of the most severe forms of sepsis, while liver dysfunction, left ventricular dysfunction, and coagulopathy impact the prognostic. Sepsis-related hypothermia and a hypoinflammatory state are related to a poor outcome. Given the heterogeneity of sepsis and recent technological progress amongst machine learning analysis techniques, a new, personalized approach to sepsis is being intensively studied. Despite the difficulties when tailoring a targeted approach, with the use of artificial intelligence-based pattern recognition, more and more publications are becoming available, highlighting novel factors that may intervene in the high heterogenicity of sepsis. This has led to the devise of a phenotypical approach in sepsis, further dividing patients based on host and trigger-related factors, clinical manifestations and progression towards organ deficiencies, dynamic prognosis algorithms, and patient trajectory in the Intensive Care Unit (ICU). Host and trigger-related factors refer to patients’ comorbidities, body mass index, age, temperature, immune response, type of bacteria and infection site. The progression to organ deficiencies refers to the individual particularities of sepsis-related multi-organ failure. Finally, the patient’s trajectory in the ICU points out the need for a better understanding of interindividual responses to various supportive therapies. This review aims to identify the main sources of variability in clustering septic patients in various clinical phenotypes as a useful clinical tool for a precision-based approach in sepsis and septic shock.

## 1. Introduction

Sepsis is defined as a life-threatening organ dysfunction caused by a dysregulated host response to infection, which in particularly severe cases can lead to multiple organ insufficiencies and septic shock, leading to higher mortality than sepsis alone. According to the third international consensus on sepsis and septic shock (Sepsis-3), sepsis is to be suspected in patients with an infection and confirmed by a Sequential Organ Failure Assessment (SOFA) score ≥ 2. Septic shock is defined as a need to introduce vasopressor therapy to maintain a patient’s mean arterial pressure (MAP) ≥ 65 mmHg, along with a serum lactate level ≥ 2 mmol/L [1].

The vast heterogeneity of interindividual clinical and paraclinical expressions, along with the globally increasing incidence and high mortality rates in both high and low-income countries, has deemed this pathology a matter of world health and has furthermore prompted the medical community to extensively research the underlying mechanisms causing this heterogeneity.

**Figure 1:**
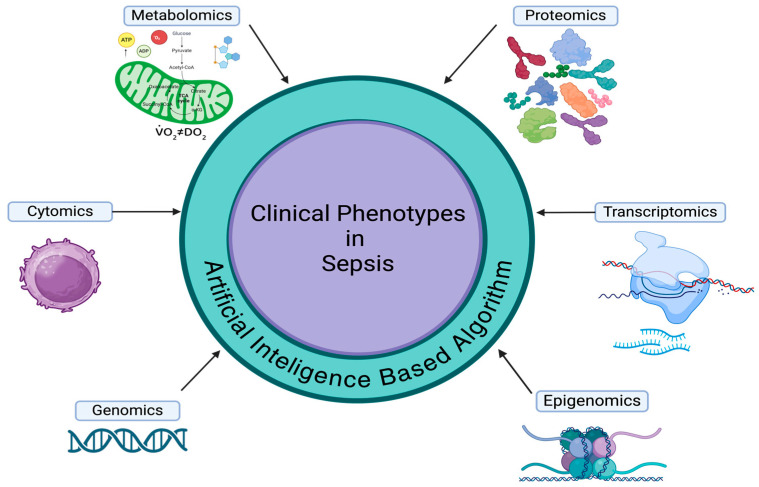
The main molecular determinants in sepsis phenotyping.

Therefore, given the recent technological advances, the medical community has begun researching part of those mechanisms at both a micro-scale, leading to the study of “Omics”, such as genomics, epigenomics, cytomics, proteomics, metabolomics, and transcriptomics, and at a macro-scale, using various proprietary algorithms and machine learning-based analysis, leading to complex classifications of septic phenotypes [2] (Figure 1).

Despite intensive research on a molecular and genetic scale, the current interest lies in refining clinical practices due to the high inter-patient variability in both prognosis and pathophysiology. Furthermore, in response to therapeutic approaches, a personalized approach is regarded as a possible solution to improving patient outcomes.

**Figure 2:**
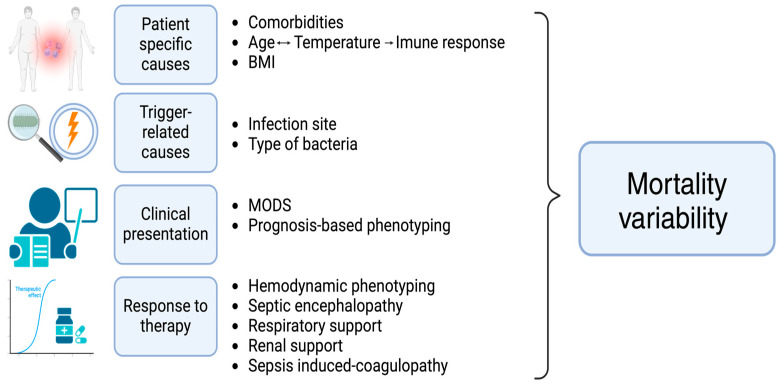
The clinical phenotyping impact on the mortality variability in sepsis.

The main objective of this review is to provide a comprehensive overview of previously described bacterial-related septic phenotypes based on four perspectives: patient-related factors, trigger-related factors, clinical manifestations and ICU trajectory, and response to various therapeutic strategies with an impact on mortality rate (Figure 2).

## 2. Patient-Specific Causes of Heterogeneity

### 2.1. Comorbidities

Given the high heterogeneity in sepsis, various studies relate the individual patient factors and comorbidities and the variability of clinical outcomes and response to various therapeutic strategies [3].

As of 2019, along with Seymour’s publication, which demonstrated that the mortality rates of older patients with renal dysfunction and those of patients with severe liver dysfunction and coagulopathy were significantly different, Ding et al. (2021) identified three distinct phenotypes, emphasizing the impact of chronic conditions such as diabetes, hypertension, and COPD on mortality rates [4,5]. 

Similarly, in the same year, an extensive paper was published evaluating post-discharge outcomes, showing that comorbidities such as chronic kidney disease, cardiovascular disease and liver dysfunction significantly influenced readmission and mortality rates among previous sepsis survivors [6]. A subsequent study reinforced these findings by identifying coagulation phenotypes with distinct clinical outcomes, determining that liver dysfunction and disseminated intravascular coagulation were closely associated with severe coagulation abnormalities [7]. Recently, DeMerle et al. divided septic patients into two main phenotypes: Phenotype 1, with more inflammation, organ dysfunctions and worse clinical outcomes and Phenotype 2, with a better clinical outcome [8]. Cumulatively, these studies highlight the impact of patient comorbidities on survival, recovery and rehabilitation rates, along with the role they might play in the progression toward certain pathophysiological trajectories (Table 1).

### 2.2. Age, Temperature and Immune Response Relationship

The relationship between age, temperature, comorbidities, and immune response in septic patients was highlighted, emphasizing the correlation between these individual variables [9,10]. According to Honore et al., elderly patients often exhibit a blunted febrile response because of reduced production of pyrogenic cytokines such as IL-6 and TNF-α. The reasoning behind it lies in reduced muscle mass, decreased heat production, and a higher incidence of comorbidities such as stroke, which may render thermoregulatory and inflammatory responses impaired [9]. Moreover, the natural immune defense mechanisms, like the microbiome, are severely impaired, especially in geriatric septic patients, which further could disturb the immune response. Conversely, the younger, septic patients with hypothermia, defined as a body temperature < 36.0 °C, had significantly increased 90-day mortality rates compared to normothermic or feverish patients. Paradoxically, in the elderly population, hypothermia was not associated with elevated mortality, which suggests a different inflammatory response as opposed to their younger counterparts. Subsequently, it is suggested that the thermoregulatory mechanisms and immune status play a significantly more important role in determining outcomes in younger patients’ hyper-inflammation, immune exhaustion, age-related immunity, and microbiome interference [11]. The exacerbated immune responses like hyperinflammation, which produces immune exhaustion, could explain some age-related differences in mortality of septic patients with thermoregulatory impairments. 

The study of Shimazui et al. further clarified the importance of the age-temperature-immune response [9]. Firstly, using multiple cohort validation, the study found that non-elderly septic patients (age < 75 years) with hypothermia had significantly increased 90-day mortality rates, in accordance with previous literature [12,13]. However, it has also been noted that in the context of fever, mortality was decreased in the non-elderly category but with no significant impact in older patients. The study also noted that comorbidities such as stroke, in which thermoregulation and inflammatory responses might be impaired, were more prevalent in the elderly group.

Both studies underscore the critical role of age in modulating the relationship between temperature, immune response, and mortality in septic patients, which has prompted the hypothesis and, furthermore, the development of studies on the topic of induced hyperthermia in sepsis. Table 2 reveals the main studies focusing on the relationship between age, temperature and immune response.

### 2.3. Body Mass Index

The relationship between body mass index (BMI), obesity, and sepsis phenotypes has been a significant point of focus for recent research, particularly correlating these factors to mortality rates and clinical outcomes [14,15].

Seymour et al. (2019) confirmed a correlation between BMI and sepsis phenotypes. This study identifies four clinical sepsis phenotypes (α, β, γ, and δ) based on demographics, laboratory values and organ dysfunction patterns, with specific comorbidities associated with each phenotype. The δ phenotype, associated with the highest mortality rates, was notably linked to elevated BMI levels. This study found that higher BMI was a significant factor in determining the severity and outcomes of sepsis. The findings of Seymour et al. suggested that patients with higher BMI may experience worse clinical outcomes due to the complex interplay between adiposity, inflammation, and immune response [4].

Conversely, the “obesity paradox” in sepsis, observed in several studies, adds another layer of complexity to the relationship between BMI and the sepsis patient’s outcomes [11]. This paradox advocates that higher BMI may be associated with lower mortality in critically ill patients, potentially due to the protective role of adipose tissue in supporting immunologic functions and thermogenesis [15]. Adipose tissue may provide a metabolic reserve that helps sustain the body’s immune response during the acute phase of sepsis. Furthermore, low BMI is associated with increased mortality, likely due to weaker immunity and lower thermogenic capacity, impairing the body’s ability to effectively respond to infection [11,16]. Thus, a nuanced understanding of how BMI and adiposity influence sepsis outcomes may be an important factor to consider in clinical decision-making.

Ito et al. provided valuable insight into the interaction between body temperature, BMI, and mortality in septic patients. The study demonstrated that hypothermia was associated with higher in-hospital mortality only in patients within the normal BMI range, suggesting that the normal BMI group is particularly vulnerable to the adverse effects of hypothermia, emphasizing the need for careful monitoring and management of body temperature in this subpopulation. The significant association between hypothermia and mortality in the normal BMI group highlights the critical role of BMI; thus, considering the BMI in regard to phenotypic classifications of sepsis might aid in better prediction of patient outcomes and guide treatment strategies accordingly [11].

## 3. Trigger-Related Causes of Heterogeneity

### 3.1. Infection Site

The influence of the site of infection in sepsis phenotyping is a crucial aspect of sepsis research, carrying significant implications for patient management and treatment outcomes. Various studies highlight the significant impact of pulmonary infections on the mortality and long-term outcomes of sepsis patients [17,18,19].

According to Jeganathan et al., pulmonary infections had the highest mortality rate (28%) among various infection sites, with abdominal infections presenting an intermediate mortality rate (15%) and genitourinary infections the lowest (7%) [20]. In contrast, Jeganathan et al. conducted a prospective longitudinal study exploring phenotypic heterogeneity by the site of infection in surgical sepsis. The study revealed that patients with abdominal infections had higher rates of organ dysfunction and mortality compared to those with infections at other sites. Specifically, abdominal infections were associated with a 28-day mortality rate of 30.65%, significantly higher than other infection sites [17].

Chen et al.’s extensive study in 2020, which spanned 11 years and included a cohort of 1,259,578 sepsis patients, revealed that lower respiratory tract infections were the most prevalent causes of sepsis and were associated with the highest mortality rates. This large-scale analysis underscores the critical burden pulmonary infections impose on sepsis outcomes, emphasizing the need for targeted interventions to effectively manage and mitigate these infections [18].

In 2021, He et al. conducted a study involving 483 sepsis patients, providing a detailed breakdown of infection sites. They found that lung infections accounted for 56.3% of cases, abdominal infections for 37.3%, and infections at other sites for 6.4%. Importantly, this study identified pulmonary infections as independent risk factors for both mortality and poor quality of life one year after sepsis. This finding highlights the long-term detrimental effects of pulmonary infections on sepsis survivors, further emphasizing the importance of early and effective management of these infections to improve long-term outcomes [19].

A study by Chou et al. analyzed the hospitalization data of 7,860,687 adult sepsis patients, categorizing infection sites into various systems such as urinary and reproductive, lower respiratory tract, circulatory, skin, abdomen, catheter, musculoskeletal, biliary tract, and others. The study revealed that the top three highest mortality rates were associated with abdominal infections (30.65%), lower respiratory tract infections (27.70%), and biliary tract infections (25.48%). These findings underscore the critical role of the infection site in determining sepsis outcomes and highlight the need for specific management strategies for high-risk infection sites [21]. 

Papin et al. retrospectively analyzed 6046 ICU-admitted sepsis patients and identified six phenotypic clusters with distinct characteristics and outcomes. For instance, Phenotypes 1 and 2 included young patients without comorbidities admitted for community-acquired pneumonia or meningitis/encephalitis, respectively. In contrast, Phenotypes 3 and 4 comprised older adults with chronic obstructive pulmonary disease, bronchial infection, and varying degrees of organ failures and comorbidities. Phenotype 5 represented postoperative patients with nosocomial infections, while Phenotype 6 included young, immunosuppressed patients. Mortality rates at 28 days, 90 days, and one year varied significantly across these phenotypes, highlighting the impact of the infection site and patient characteristics on sepsis outcomes [22].

Most recently, Schertz et al. conducted a retrospective cohort study involving 6753 hospitalized adults to identify clinical sepsis phenotypes based on the site of infection. The study found significant demographic, vital sign, and laboratory result differences across various infection sites [23]. Notably, unknown or unspecified infections had the highest proportion of shock or 30-day mortality (21.34%), while CNS infections had the lowest (8.11%). Respiratory, vascular, and unknown or unspecified infection sites demonstrated significantly increased odds of adverse outcomes compared to other sites [24].

The relationship between the site of infection and septic phenotypes is pivotal for developing targeted and effective treatment strategies. By understanding the specific characteristics and risks associated with different infection sites, healthcare providers can better predict patient outcomes and tailor interventions accordingly.

### 3.2. Type of Bacteria

The relationship between the type of bacteria and mortality in septic patients is of paramount importance [24]. For instance, a study by Falcone et al. investigated the impact of carbapenem-resistant Gram-negative bacilli on mortality, finding that bloodstream infections caused by these pathogens were associated with significantly higher mortality rates compared to infections caused by carbapenem-susceptible strains [25]. Similarly, a systematic review and meta-analysis by Maraolo et al. emphasized the mortality attributable to bloodstream infections caused by different carbapenem-resistant Gram-negative bacilli, reinforcing the critical impact of bacterial type on sepsis outcomes [26]. A longitudinal analysis over a 20-year period in a neonatal intensive care unit, observing trends in incidence, pathogens, and mortality. Their findings indicated that healthcare-associated bloodstream infections, particularly those caused by Gram-negative bacteria, were linked to higher mortality rates [27]. Another study by Arvaniti et al. focused on critically ill patients with intra-abdominal infections, revealing that the type of bacterial pathogen significantly influenced age-related mortality outcomes [28].

The study by Papin et al. utilized hierarchical clustering to identify clinical and biological clusters of sepsis patients, noting that infections with non-fermentative Gram-negative bacilli and Staphylococcus aureus were associated with distinct clinical outcomes and higher mortality rates [22]. 

In summary, these studies collectively reveal the profound impact of bacterial type on sepsis mortality, highlighting the need for targeted therapeutic strategies. The consistent finding that Gram-negative bacteria, particularly carbapenem-resistant strains, are associated with higher mortality rates underscores the urgency of developing effective treatments and interventions tailored to the specific bacterial etiology in septic patients.

## 4. Clinical Manifestations and Prognosis-Based Phenotyping

### 4.1. Multiorgan Dysfunction Phenotyping

The study of machine learning systems in sepsis clustering with regard to multiple organ dysfunction syndrome promotes the phenotype-based approach [29]. Analyzing a cohort of 2533 patients diagnosed with severe sepsis or septic shock, Knox et al. [29] employed self-organizing maps (SOMs) and k-means clustering to identify four distinct phenotypic clusters based on organ dysfunction patterns. This approach was pivotal in delineating the heterogeneity inherent in sepsis-associated multiple organ dysfunction syndrome (MODS). The first cluster, termed “Shock with Elevated Creatinine,” primarily encompassed patients exhibiting pronounced kidney dysfunction, characterized by elevated creatinine levels indicative of renal failure. This group demonstrated an intermediate severity of illness with a notable prevalence of septic shock. Despite the significant renal impairment, the mortality rates were not the highest, suggesting that early and aggressive renal support could mitigate some of the associated risks.

In contrast, the “Minimal MODS” cluster included patients with minimal organ dysfunction and various mild sepsis presentations. This phenotype exhibited the lowest mortality rate. The cluster included a broad spectrum of infections, such as pneumonia and urinary tract infections, leading to better outcomes with minimal intervention. These findings suggest that meticulous monitoring and supportive care could suffice for patients within this cluster, avoiding unnecessary aggressive treatments. The “Shock with Hypoxemia and Altered Mental Status” cluster comprised patients who manifested severe respiratory distress and neurological impairments, including significant hypoxemia and altered mental status. This group recorded the highest mortality rate, reflecting the extreme severity associated with combined respiratory and neurological dysfunctions. Lastly, the “Hepatic Disease” cluster predominantly involved patients with liver dysfunction, often coupled with coagulation disorders. This phenotype was marked by significant morbidity and a mortality rate. The challenges in managing this cluster were exacerbated by the presence of acute liver injury patterns and conditions like end-stage liver disease, complicating clinical interventions due to associated high morbidity and mortality risks [29].

Regression analysis revealed that these phenotypic clusters were largely independent of age, cause of sepsis, obesity, and other comorbidities. Furthermore, the study noted significant differences in the association between clinical outcomes and predictors, such as the Acute Physiology And Chronic Health Evaluation (APACHE) II score [29]. Additionally, Ibrahim et al. undertook an extensive examination of sepsis phenotypes using data from 13,728 septic patients admitted to the ICU. By calculating 63 vital signs and laboratory tests collected within the first 24 h of admission and applying machine learning methodologies similar to those used by Knox et al. in 2015 [29], they identified four clinically significant sepsis subpopulations, each exhibiting distinct organ dysfunction patterns [30].

The first phenotype, indicative of liver disease, included patients suffering from hepatic dysfunction and coagulation disorders, and this group was associated with a 30-day mortality rate of 28%. The second phenotype, characterized by cardiogenic dysfunction with elevated creatinine levels indicative of renal impairment, exhibited the highest 30-day mortality rate at 55%. The third phenotype comprised patients with minimal organ dysfunction, marked by relatively stable vital signs and laboratory results, and this group had a 30-day mortality rate of 25%, reflecting a moderate severity of illness. The fourth phenotype involved cardiogenic dysfunction accompanied by hypoxemia and altered mental status, with a 30-day mortality rate of 37%, highlighting the critical nature of combined cardiovascular, respiratory, and neurological impairments.

Additionally, it has been observed that patients with the liver disease phenotype had prolonged hospital stays and a higher likelihood of requiring mechanical ventilation, while the cardiogenic dysfunction with elevated creatinine phenotype exhibited increased use of vasopressors, reflecting severe cardiovascular instability. The minimal organ dysfunction phenotype had shorter hospital stays and less need for intensive interventions, while the cardiogenic dysfunction with hypoxemia and altered mental status phenotype had higher rates of neurological complications and prolonged ICU stays [30].

Furthermore, Zhang et al. conducted a retrospective analysis involving 14,993 septic patients, utilizing machine learning techniques to classify these patients into four distinct phenotypic groups based on organ dysfunction patterns. This classification aimed to better understand the heterogeneity in sepsis presentations and outcomes [31].

The first phenotype, labeled as the baseline type, comprised 69% of the patients and was characterized by relatively stable clinical parameters and the lowest mortality rate. The second phenotype, which included 9% of the patients, was marked by respiratory dysfunction. Patients in this group required significant respiratory support and had a mortality rate. The third phenotype, representing 11% of the patients, was characterized by multiple organ dysfunction, including kidney, coagulation, liver, and shock. This group exhibited the highest mortality rate at 45.4%, highlighting the severe impact of multi-organ failure in sepsis. The fourth phenotype comprised 11% of the patients and was defined as neurological dysfunction. Patients in this group had significant neurological impairments, such as altered mental status, and a mortality rate of 27.4%. As part of the secondary outcomes, it has been shown that the patients in the third phenotype received the largest amount of fluid during the first 24 h, which was associated with a reduced risk of hospital mortality. In contrast, higher fluid inputs were linked to increased mortality in the fourth phenotype [31].

Seymour et al. identified four unique sepsis phenotypes, labeled α, β, γ, and δ [4]. Notably, the δ phenotype, which included 13% of the patients, was associated with elevated serum lactate levels, increased transaminases and hypotension, signaling severe metabolic and hepatic dysfunction, and consequently exhibited the highest 28-day mortality rate at 40%. Patients classified within the δ phenotype required the most intensive care, exemplified by prolonged mechanical ventilation and higher doses of vasopressors, reflecting the severity of their clinical condition. Conversely, patients within the α phenotype experienced shorter hospital stays and necessitated fewer intensive interventions, underscoring the relatively benign nature of their clinical presentations in comparison to the other phenotypic groups [4].

In the study conducted by Xu et al., researchers delved into the intricate dynamics of sepsis by examining organ dysfunction trajectories to identify distinct sepsis phenotypes, using 72 h SOFA score assessments. Utilizing data from a cohort of 16,743 patients, the study employed group-based trajectory modeling to classify patients into four unique phenotypes: Rapidly Worsening, Delayed Worsening, Rapidly Improving, and Delayed Improving [32].

The Rapidly Worsening group exhibited the most severe clinical deterioration, characterized by significant derangements in metabolic acidosis and hypoperfusion markers, such as low bicarbonate and high lactate levels. Additionally, these patients showed signs of disseminated intravascular coagulation, including low platelet counts and high INR, alongside respiratory failure. This group had a high comorbidity burden, which was a strong predictor of their rapid decline in organ function. In contrast, the Delayed Worsening group experienced a slower progression of organ dysfunction, exhibiting a mix of hematologic, cardiovascular, and renal abnormalities. Although this group also had a significant comorbidity burden, it was less severe than the Rapidly Worsening group. The Rapidly Improving phenotype was marked by a swift recovery in organ function. Patients in this group had more abnormal inflammatory markers at ICU admission, such as elevated WBC counts, bands, and abnormal albumin levels. They were more likely to have urosepsis and showed abnormal cardiovascular and CNS deteriorations. These patients had a lower comorbidity score, suggesting that their sepsis outcomes were more dependent on the acute illness rather than underlying chronic conditions. The Delayed Improving group showed a gradual improvement in organ function. Their clinical variables were less specific but included inflammatory, hepatic, hematologic, and pulmonary markers. Similar to the Rapidly Improving group, these patients had a lower comorbidity burden. The Rapidly Worsening group had the highest in-hospital mortality at 28.3%, despite having a lower initial SOFA score compared to the Rapidly Improving group, which had a mortality rate of 5.5% [32]. 

In their study, Ding and Luo classified sepsis phenotypes based on organ dysfunction by utilizing nonnegative matrix factorization (NMF) of temporal trends from a multivariate panel of physiological measurements [5]. Drawing from the Medical Information Mart for Intensive Care III (MIMIC-III) database, which houses de-identified electronic health records from over 60,000 ICU stays, they selected a cohort of 5782 patients whose sepsis onset coincided with their ICU admission. Researchers identified three novel phenotypes distinguished by their clinical characteristics and prognostic implications. Subgroup 1, encompassing 21% of the cohort (*n* = 1218), included relatively less severe cases with a 30-day mortality rate of 17%. This group was characterized by an older mean age of 73 years, a male majority (male-to-female ratio of 59-to-41), and complex chronic conditions. Subgroup 2, the second largest at 35% (*n* = 2036), represented the most severe cases with a 30-day mortality rate of 28%. Patients in this group exhibited severe organ dysfunction or failure, compounded by a wide range of comorbidities, and had notably high incidences of coagulopathy and liver disease. Subgroup 3, the largest at 44% (*n* = 2528), included the least severe cases with a 30-day mortality rate of 10%. This group was characterized by a younger mean age of 60 years, a balanced gender ratio (male-to-female ratio of 50-to-50), the least complicated conditions, and a uniquely high incidence of neurological disease. The high mortality rate from Subgroup 2 highlighted the severe impact of combined organ dysfunctions, particularly liver disease and coagulopathy [5].

Complementarily, Aldewereld et al. performed an in-depth analysis of septic shock phenotypes using a cohort of 1023 subjects from the ProCESS trial [33]. The study revealed five phenotypes, each with unique organ failure patterns and varying degrees of illness severity. These phenotypes were classified into two low-risk groups (L1 and L2), one moderate-risk group (M), and two high-risk groups (H1 and H2). The first phenotype, L1, was characterized as a fluid-refractory shock without multi-organ dysfunction. This group, which comprised 28% of the subjects, had relatively low illness severity scores and showed moderate vasopressor support needs with minimal respiratory support. The 14-day and 60-day mortality rates for this group were 7.9% and 14.6%, respectively. The second phenotype, L2, was identified as fluid-responsive shock and included 21% of the subjects. This younger group exhibited low illness severity scores and a low incidence of bacteremia. The 14-day and 60-day mortality rates were 12.6% and 17%, respectively. 

The moderate-risk phenotype, M, was predominantly characterized by respiratory failure and included 16% of the subjects. This group had 14-day and 60-day mortality rates of 25.5% and 34.3%, respectively. The incidence of pneumonia was highest in this group, and although they had high APACHE scores, their cardiovascular and renal dysfunctions were less severe compared to the high-risk groups. High-risk phenotypes H1 and H2, which included 19% and 16% of the subjects, respectively, were associated with the highest mortality rates. The H1 phenotype was defined by multiple organ dysfunctions, including severe cardiac and respiratory failures, with 14-day and 60-day mortality rates of 28.8% and 42.4%, respectively. This group exhibited the highest APACHE and SOFA scores, along with significant vasopressor and mechanical ventilation requirements. The H2 phenotype, characterized by liver dysfunction and coagulopathy, had 14-day and 60-day mortality rates of 36.3% and 44.1%, respectively. This group was unique for its high incidence of intra-abdominal infections and positive blood cultures, along with pronounced lactate elevation and significant platelet count and bilirubin abnormalities [33].

Using an AI-based technique, Sharafoddini et al. identified 12 phenotypes among 5539 adult septic patients [34]. The study found significant variability in mortality rates across these phenotypes, with clusters 10, 11, and 8 showing the lowest mortality rates and clusters 9 and 2 showing the highest. Furthermore, the clusters with the highest mortality rates also showed a higher prevalence of severe organ dysfunctions and a greater need for intensive care interventions such as mechanical ventilation and vasopressor support [34]. Table 3 highlights the main conclusions of various studies focusing on sepsis clustering based on the presence of multi-organ dysfunction.

A consistent finding across these studies was the significant variability in primary outcomes, particularly mortality rates, among the different phenotypes. High mortality rates were uniformly associated with phenotypes exhibiting severe multi-organ dysfunction. Secondary outcomes, including the need for mechanical ventilation, vasopressor support, and length of hospital stay, further highlighted the severe clinical burden borne by these high-risk phenotypes [4,32]. 

Interestingly, the studies revealed discrepancies in the specific organ dysfunctions emphasized and the methodologies employed. For instance, Ibrahim et al. [30] focused on cardiogenic dysfunction and liver disease, while Ding and Luo (2021) highlighted neurological disease and coagulopathy [5]. These variations could potentially influence the reproducibility and applicability of the findings.

While the studies collectively highlight the paramount importance of phenotypic classification in sepsis management, the observed discrepancies highlight the necessity for standardization. By identifying distinct sepsis phenotypes with significant differences in clinical outcomes, these studies pave the way for more targeted and effective therapeutic strategies, ultimately aiming to improve patient outcomes in the complex landscape of sepsis management.

### 4.2. Prognosis-Based Phenotyping

The prognostic in sepsis and septic shock relies on various scoring systems used, including SOFA and APACHE scores [35]. Moreover, various prognostic biomarkers are extensively studied: procalcitonin, presepsin, CD64, suPAR, and sTREM-1. The research by Xu, Zhang, and Yang collectively underscores the pivotal role of SOFA score trajectories in delineating sepsis phenotypes and prognosticating clinical outcomes, yet they reveal notable similarities and discrepancies in their findings [32,36,37].

All three studies utilized advanced statistical methodologies to classify patients into distinct SOFA score trajectory groups, emphasizing the dynamic nature of organ dysfunction in sepsis and the critical importance of continuous scoring. The prognosis-based phenotyping originates from the dynamics of various scoring systems used. 

A salient similarity across the studies is the identification of high-risk phenotypes characterized by persistently elevated SOFA scores, which were consistently associated with the poorest prognoses and highest mortality rates [32,36]. Xu et al. identified the Rapidly Worsening phenotype, marked by continuously increasing SOFA scores and a 30-day mortality rate of 28.3%, while Zhang et al. described Class 2 and Class 5, both exhibiting high initial SOFA scores and high mortality rates of 70% and 41.2%, respectively. Similarly, Yang et al. observed in the Class 5 group, with persistently high SOFA scores, the highest cumulative risk and the poorest outcomes, including a 7-day in-hospital mortality [32,36,37].

Despite these common findings, discrepancies emerge in the trajectory patterns and their implications for clinical management. Xu et al. highlighted the prognostic value of early improvement in SOFA scores, with the Rapidly Improving phenotype demonstrating a swift recovery and a significantly lower 30-day mortality rate of 5.5% [32]. In contrast, Zhang et al. identified a moderate initial SOFA score trajectory in Class 3, followed by a decreasing severity during the ICU stay, representing the largest class with 51.7% of subjects and a more favorable prognosis [36]. Yang et al. further pointed out that even moderate increases in SOFA scores, as seen in Groups 2 and 3, were independently associated with increased risks of mortality and adverse outcomes, underscoring the importance of timely interventions to mitigate these risks [37]. The studies also differ in their secondary outcome measures and the statistical significance of these outcomes. Xu et al. emphasized the need for renal replacement therapy, mechanical ventilation, and vasopressor use, with the Rapidly Worsening group requiring the most intensive interventions [32]. Zhang et al. highlighted the transition to persistent critical illness (PCI) and the elevated urea-to-creatinine ratio as significant biochemical markers, while Yang et al. focused on the incidence of septic shock and acute respiratory failure (ARF), with Group 5 patients being at the highest risk [36,37]. These differences in secondary outcomes reflect the varied clinical presentations and management challenges associated with different SOFA score trajectories.

In conclusion, the SOFA score trajectory is of critical importance in understanding sepsis phenotypes and prognosticating outcomes. The urge for personalized and timely therapeutic strategies tailored to the specific SOFA score trajectories, ultimately aiming to improve patient outcomes in the complex landscape of sepsis management, could represent a daily clinical tool for intensive care clinicians.

## 5. Organ Dysfunction Trajectory and Response to the Therapeutic Approach in Clinical Sepsis Phenotyping

### 5.1. Hemodynamic Phenotyping

**Figure 3:**
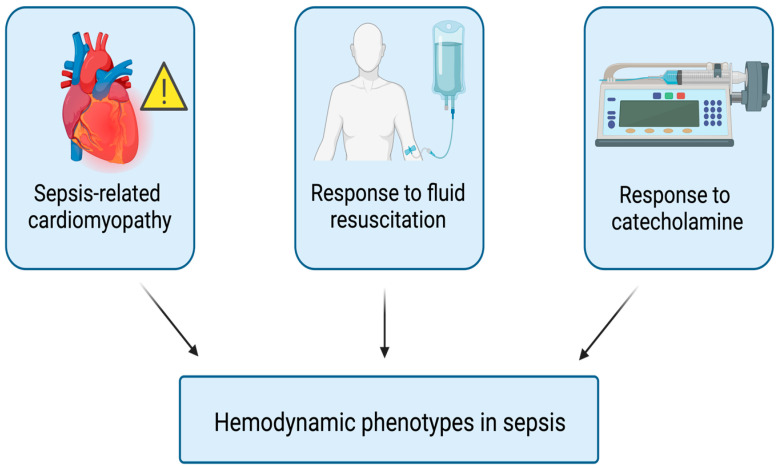
Hemodynamic phenotypes in sepsis.

Septic shock, characterized by hemodynamic instability leading to multi-organ dysfunction and potentially fatal outcomes, requires a nuanced understanding of hemodynamic phenotypes to enhance targeted therapeutic strategies. There are three main factors that affect the hemodynamic variability in sepsis and septic shock: sepsis-related cardiomyopathy, response to fluid resuscitation, and differentiated response to catecholamines (Figure 3).

Regarding sepsis-related cardiomyopathy, Geri et al. conducted a comprehensive study analyzing 360 intensive care patients using a machine learning algorithm to identify five distinct hemodynamic profiles within a septic context [38]. Each cluster demonstrated significant variations in mortality rates. Cluster 1, consisting of 61 patients (16.9%), showed no left ventricular (LV) systolic dysfunction, right ventricular (RV) failure, or fluid responsiveness and had the lowest mortality rates (9.8% on Day 7 and 21.3% overall ICU mortality), indicating relative hemodynamic stability. Cluster 2, with 64 patients (17.7%), was characterized by LV systolic dysfunction and had significantly higher mortality rates (32.8% on Day 7 and 50.0% in the ICU). Cluster 3, comprising 84 patients (23.3%), exhibited a hyperkinetic profile with high cardiac output and lower mortality rates (8.3% on Day 7 and 23.8% in the ICU), likely due to early and effective interventions. Cluster 4 included 81 patients (22.5%) with RV failure, showing mortality rates of 27.2% on Day 7 and 42.0% in the ICU, highlighting the severe impact of RV failure on prognosis. Cluster 5, encompassing 70 patients (19.4%) with persistent hypovolemia, had Day 7 and ICU mortality rates of 23.2% and 38.6%, respectively, due to inadequate tissue perfusion and organ failure [38].

Zhang et al. emphasized the classification of septic myocardial suppression into phenotypes—LV systolic dysfunction (LVSD), LV diastolic dysfunction (LVDD), right ventricular dysfunction (RVD), and mixed ventricular dysfunction—using echocardiography to tailor treatments and improve outcomes [39]. The study sought to correlate these phenotypes with outcomes such as mortality, echocardiographic improvement, ICU stay length, and mechanical ventilation duration, challenging the conventional view of irreversible myocardial suppression. The hypothesis posited that certain phenotypes, particularly those indicating reversible suppression, might be protective, allowing for full recovery if managed appropriately [39].

The ANDROMEDA-SHOCK-2 study assessed the efficacy of capillary refill time (CRT)-targeted resuscitation in septic shock [40]. This multicenter trial incorporated hemodynamic variables, including fluid responsiveness and myocardial function via echocardiography, to determine if CRT-targeted resuscitation could reduce mortality and hospital stay. By personalizing resuscitation based on CRT, this study aimed to minimize fluid overload and optimize vasoactive drug usage, refining treatment protocols according to specific cardiovascular dynamics and improving outcomes [40]. 

Additionally, Zhu et al. focused on the systolic blood pressure variability in patients clustering [41]. They identified seven distinct systolic blood pressure (SBP) trajectories via trajectory analysis. Phenotype 1 (36.9%) exhibited stable SBP at 100 mmHg, while Phenotype 2 (7.5%) maintained a stable SBP with a mean of 83 mmHg. Phenotypes 3 (8.4%) and 4 (21.3%) showed increasing SBP from 140 mmHg and 110–120 mmHg to 120–130 mmHg, respectively. Conversely, Phenotypes 5 (15.3%) and 6 (8.2%) demonstrated rapid SBP declines from 130 mmHg to 100 mmHg and 150–160 mmHg to 110–120 mmHg, respectively. Phenotype 7 (2.8%) displayed an initial SBP rise followed by a decrease [40]. Their in-hospital mortality rates were 25.5%, 40.5%, 11.8%, 18.3%, 23.5%, 13.8%, and 10.5%, respectively. Notably, Phenotype 3 exhibited the lowest mortality risk, suggesting it as a potentially optimal hemodynamic target within the first 10 h of ICU admission. A persistently low SBP (Phenotype 2) forecasted a worse prognosis than a sharp SBP decline (Phenotype 6), emphasizing the importance of monitoring SBP trends to identify high-risk patients [41]. 

The identification and understanding of hemodynamic phenotypes in sepsis are crucial for improving patient outcomes. A cluster-based approach focusing on sepsis-induced myocardial and vascular disturbances provides valuable insights into the diverse physiological responses to sepsis, enabling more precise and personalized therapeutic strategies. 

Secondly, the variability of the response to fluid resuscitation in septic shock impacts the patient’s outcome and therapy approach. The actual guidelines in sepsis and septic shock highlight a new perspective for fluid resuscitation: the individual fluid tolerance and responsiveness, by mainly ultrasonographical-based approach [42]. Clustering these patients could be helpful in a precision-based therapeutic algorithm. In 2021, Shald et al. present a detailed analysis of 320 septic patients admitted to the ICU as a retrospective single-center cohort study classifying them into four clinical phenotypes: multi-organ failure (MOF), respiratory dysfunction (RD), neurologic dysfunction (ND), and other patients (OP) [43]. The primary outcome was in-hospital mortality, revealing significant differences among the phenotypes: MOF had the highest mortality rate at 48.4%, followed by ND at 39.7%, RD at 20.8%, and OP at 13.7%. Additionally, the study found notable secondary outcomes in terms of fluid balance. Differences in volume balances between phenotypes were evident over time (48 to 96 h post-admission). Patients in the MOF and ND phenotypes had the largest positive volume balances at these time points. Furthermore, the study identified the MOF and ND phenotypes, along with volume balance at 24 h, as significant predictors of in-hospital mortality [43].

The study by Ma et al. identifies five distinct septic shock phenotypes using finite mixture modeling and K-means clustering in a cohort of 1437 patients with a 29% mortality rate [44]. The described phenotypes are: Class 1 (Baseline Class), which remained stable and transitioned to a de-resuscitation phase by day 3; Class 2 (Critical Class), with the highest severity and mortality, benefited from delayed norepinephrine use post-adequate fluid resuscitation; Class 3 (Renal Dysfunction), exhibiting significant kidney issues and requiring rapid de-resuscitation by day 1 to avoid fluid overload; Class 4 (Respiratory Failure Class), marked by pronounced respiratory failure necessitating careful fluid management to prevent lung injury exacerbation; and Class 5 (Mild Class) with the lowest mortality rate at 21%, representing patients with relatively mild symptoms and better overall outcomes [44]. Primary outcomes showed significant mortality rate variations among the classes, demonstrating that tailored resuscitative efforts, including optimal fluid volume and norepinephrine dosing, positively impacted survival rates. Secondary outcomes emphasized optimal fluid and norepinephrine administration with early, large-volume fluid resuscitation followed by restricted volumes, particularly benefiting baseline and renal dysfunction classes. Delayed norepinephrine was beneficial for the critical class, whereas early norepinephrine was advantageous for baseline and renal dysfunction classes. The study also highlighted avoiding fluid and norepinephrine overdoses by identifying risk factors such as body weight and PaCO2 levels [44]. 

The exploration of sepsis phenotypes and their response to fluid resuscitation reveals several overarching similarities across the studies reviewed. A notable commonality is the utilization of advanced machine learning and statistical methodologies to delineate distinct sepsis phenotypes, underscoring the critical role of sophisticated analytical tools in enhancing our understanding of sepsis heterogeneity [31,33].

A recurrent theme is the elevated mortality rates consistently associated with fluid refractory phenotypes. For instance, Zhang et al.’s study identified a phenotype characterized by multiple organ dysfunction, which exhibited the highest mortality rate at 45.4% and involved patients receiving the largest fluid volumes during the first 24 h [31]. Similarly, Aldewereld et al. identified high-risk phenotypes H1 and H2, which necessitated significant vasopressor and mechanical ventilation support, reflecting their fluid refractory nature and concomitant high mortality rates [33]. This pattern reveals the severe clinical burden borne by fluid refractory phenotypes. Furthermore, fluid refractory phenotypes were uniformly associated with severe organ dysfunctions across the studies [4,5,45].

Finally, patients with distinct phenotypic characteristics exhibit varying requirements for vasopressors, which has significant implications for their management and outcomes. The first-line treatment used for hypotension in sepsis is noradrenaline [46]. Actual guidelines promote the use of other medication as well: vasopressin, Methylene blue, cortisone, or various inotropic agents [47,48]. The unique combination of various substances highlights the need for a personalized approach in septic shock. Phenotypes with the least requirements for vasopressors, such as the α phenotype identified by Seymour et al., are characterized by fewer abnormal laboratory values, less organ system dysfunction, and relatively stable clinical parameters [4]. These patients typically have lower mortality rates and shorter hospital stays, reflecting a more benign clinical course. Similarly, Bhavani et al.’s Group C phenotype, marked by normothermia and normotension, also exhibited lower vasopressor use and better outcomes [45].

In contrast, phenotypes with the most significant requirements for vasopressors, such as the δ phenotype, are associated with severe metabolic and hepatic dysfunction, high serum lactate levels, and elevated transaminases [4]. These patients exhibit the highest mortality rates and require intensive care, including prolonged mechanical ventilation and higher doses of vasopressors. Bhavani et al. described in their study that the Group D phenotype, characterized by hypothermia and hypotension, needs high vasopressor doses with significantly higher 30-day mortality [45]. Geri et al.’s hemodynamic phenotypes, particularly those with left ventricular systolic dysfunction and right ventricular failure, further highlight the critical need for individualized hemodynamic support in these high-risk groups [38].

The studies by Aldewereld et al. and Zhu et al. reinforce the variability in vasopressor requirements across different sepsis phenotypes, emphasizing the necessity for phenotype-specific strategies to optimize outcomes. High-risk phenotypes with multi-organ failure patterns and persistent hypotension require more aggressive vasopressor therapy and have poorer prognoses [33,41].

Phenotypic classification provides a valuable framework for tailoring the hemodynamic profile of each septic patient (Table 4).

By identifying distinct sepsis phenotypes with varying sepsis-related cardiomyopathy, fluid resuscitation therapy, or vasopressor requirements, clinicians can implement more personalized and effective therapeutic interventions, ultimately improving patient outcomes and guiding future research and clinical trials.

### 5.2. Sepsis-Associated Encephalopathy (SAE)

SAE is a complex condition characterized by the dysfunction of the central nervous system during sepsis, manifesting in a range of symptoms from mild delirium to deep coma. The pathophysiology of SAE is multifactorial, involving a combination of neuroinflammation, ischemia, and cellular metabolic stress [49]. Neuroinflammation is a critical component initiated by systemic inflammation that triggers the activation of microglia, the brain’s resident immune cells [50,51].

Upon activation, microglia undergo morphological changes and release pro-inflammatory cytokines such as gamma interferon and tumor necrosis factor-alpha, contributing to neuronal excitability and excitotoxicity, further leading to cognitive decline and increased mortality.

Astrocytes, the most numerous cells in the brain, also play a significant role in SAE. These cells maintain brain homeostasis by regulating blood–brain barrier (BBB) permeability, brain water balance, and microcirculatory cerebral blood flow. During sepsis, astrocyte dysfunction can lead to BBB disruption, exacerbating neuroinflammation and neuronal damage [49].

Septic shock can cause cerebral hypoperfusion, leading to ischemic brain damage. Furthermore, septic shock can lead to microcirculatory impairment, characterized by neurovascular uncoupling and coagulation cascade activation, further exacerbating brain injury. Mitochondrial dysfunction, resulting from oxidative stress, impairs ATP production and increases the generation of reactive oxygen species, promoting neuronal apoptosis and functional impairment [49].

Clinically, SAE is diagnosed based on changes in mental status, ranging from delirium to coma, often assessed using standardized scales such as the Glasgow Coma Scale (GCS). Electroencephalography (EEG) is commonly used to detect changes in brain activity, with SAE typically presenting as background slowing or the presence of epileptiform discharges. MRI studies have revealed white matter hyperdensities and ischemic strokes in septic patients with acute brain dysfunction. However, the sensitivity and specificity of these findings can vary, necessitating further research to optimize imaging protocols for SAE diagnosis [49].

Biomarkers also play a role in the investigation of SAE. Markers of systemic inflammation, such as C-reactive protein and procalcitonin, can be associated with the occurrence of SAE. Additionally, biomarkers of brain damage, including protein S100b and neuron-specific enolase (NSE) or other epigenetic markers, can provide insights into the extent of neuronal injury [50,51]. Pharmacological treatments targeting neuroinflammation and microglial activation have shown promise in experimental studies but have yet to be validated in clinical trials. For instance, minocycline, cholinergic inhibitors, and vagal nerve stimulation have demonstrated potential in reducing microglial activation and neuroinflammation. However, clinical trials evaluating these interventions, such as the use of rivastigmine, have been inconclusive or shown adverse effects, underscoring the challenges of translating experimental findings into clinical practice [52].

The study by Lu X et al. on sepsis-associated encephalopathy (SAE) provides a significant contribution to the understanding of septic phenotypes, their prognostic implications, and potential treatment strategies. The study identified four distinct clinical phenotypes of SAE: ischemic-hypoxic, metabolic, mixed (ischemic-hypoxic and metabolic), and unclassified. The identification of these phenotypes was based on specific clinical criteria and hypothesized pathophysiologic mechanisms [53]. For instance, the ischemic-hypoxic phenotype was characterized by hypoxemia or septic shock, while the metabolic phenotype was defined by metabolic disturbances such as hyperammonemia, hypoglycemia, or significant hepatic dysfunction. The findings revealed that each phenotype has distinct prognostic implications. The mixed phenotype, which combines features of both ischemic-hypoxic and metabolic dysfunctions, was associated with the highest in-hospital mortality rate and longer hospital stays [53]. This high mortality rate for the mixed phenotype suggests severe organ failure and a more complex pathophysiologic process, which could involve both inadequate cerebral perfusion and metabolic toxicity.

Dexmedetomidine, an alpha-2 adrenergic agonist, has been shown to increase delirium-free days and reduce 28-day mortality in septic shock patients [49,54]. However, recent large studies have not demonstrated significant benefits of early goal-directed sedation with dexmedetomidine in critically ill patients [55]. The potential neuroprotective effects of other agents, such as ketamine and sevoflurane, are currently being investigated in clinical trials. Since little advances have been made with regard to SAE-related clustering of patients, future research is needed to confirm its role in the precision-based medicine approach to sepsis and septic shock.

### 5.3. Respiratory Trajectory

Respiratory failure is a critical component of ARDS and sepsis, leading to significant morbidity and mortality. This feature is even more complex in immunosuppressed patients [3]. The study by Shankar-Hari et al. identifies distinct phenotypes within ARDS and sepsis populations, which exhibit varying degrees of respiratory failure. The analysis of the HARP-2 trial, which focused on ARDS, revealed two phenotypes: the hyperinflammatory and hypoinflammatory phenotypes [56]. The hyperinflammatory phenotype was associated with more severe respiratory failure, characterized by higher levels of inflammatory biomarkers such as IL-6, IL-8, and TNFR1, along with more acidosis and a greater prevalence of shock. These patients required more intensive respiratory support, including mechanical ventilation [56]. The biomarkers were not only indicative of the severity of the condition but also predictive of the clinical outcomes, with higher levels correlating with worse prognosis and higher mortality. 

The study also reveals the importance of non-pharmacological interventions in managing respiratory failure in ARDS and sepsis. Strategies such as optimizing mechanical ventilation settings, using prone positioning, and implementing lung-protective ventilation protocols are crucial in managing patients with severe respiratory failure [56].

Similarly, Sinha et al. describe the same two phenotypes, hypo- and hyperinflammatory. As part of the study, the importance of personalized interventions is highly underlined, such as fluid management and positive end-expiratory pressure (PEEP), which may have different impacts depending on the patient’s phenotype. This is particularly relevant as the hyperinflammatory phenotype tends to have worse outcomes with standard fluid management strategies, indicating a need for more tailored approaches [57]. Briefly, integrating the two main clinical forms of sepsis-related respiratory phenotypes into a more complex secondary multi-organ dysfunction pattern could influence the clinical decision based on a specific precision-based medicine algorithm.

### 5.4. Renal Injury and Renal Replacement Therapy (RRT)

Sepsis-related kidney injury is one of the most frequent complications [58]. A more personalized approach to the septic patient with renal impairment could impact his outcome [59]. Utilizing latent class analysis, Wiersema et al. identified two distinct phenotypes of sepsis-related acute kidney injury (AKI) based on 30 variables obtained upon admission to the ICU [60]. Phenotype 1 included 133 patients (44%), while Phenotype 2 comprised 168 patients (56%). Phenotype 2 was characterized by higher levels of inflammatory and endothelial injury markers, such as heparin-binding protein (HBP), neutrophil elastase 2 (Ela), proteinase 3 (PRTN3), and matrix metalloproteinase 8 (MMP8), and clinically, by patients with a lower body mass index (BMI), higher vasopressor requirements, and higher fluid balance at 72 h compared to those in Phenotype 1. Subsequently, Phenotype 2 was associated with an increased risk of 90-day mortality and decreased short-term renal recovery on day 5, suggesting a much more severe form of septic AKI [60]. 130 The study also highlights the differential need for renal replacement therapy (RRT) among the phenotypes, with Phenotype 2 having a likelihood to receive RRT of 39.3%, as opposed to 23.3% for Phenotype 1.

Other studies revealed three phenotypes according to clinical and biomarkers trajectory, grouped as early reversal, persistent AKI and relapsed AKI [61]. Early reversal was defined by the resolution of AKI within seven days, characterized by a return to normal kidney function and favorable long-term outcomes, persistent AKI, containing patients with ongoing kidney dysfunction beyond seven days, poorer clinical outcomes and a higher likelihood of progression to chronic kidney disease (CKD) and relapsed AKI, defined by an initial reversal of AKI followed by subsequent episodes of renal dysfunction, indicating an unstable clinical course and increased risk of long-term complications [61]. The study explores the role of RRT in managing severe AKI. It was observed that the timing of RRT initiation did not significantly affect overall mortality rates; however, early initiation of RRT was associated with improved renal recovery in some patient subgroups. The study emphasizes that RRT should be initiated based on clinical judgment and patient-specific factors, using the guideline-recommended delivery dose of 20–25 mL/kg/h [61].

### 5.5. Sepsis-Induced Coagulopathy (SIC)

SIC is a critical complication characterized by a dysregulated coagulation cascade. The pathophysiology of SIC involves a delicate balance between procoagulant and anticoagulant pathways that become disrupted in the context of sepsis, leading to systemic thrombin generation, impaired anticoagulant activity, and suppression of fibrinolysis [62]. Key mechanisms include increased procoagulant tissue factor (TF) expression on monocytes and endothelial cells and the formation of neutrophil extracellular traps (NETs), which promote thrombin generation and marked vascular inflammation with endothelial injury. Additionally, the impaired activity of natural anticoagulants such as antithrombin (ATIII) and protein C further exacerbates the coagulation disorder, resulting in microvascular thrombi, which can progress to organ dysfunction [62]. 

As of 2021, Kudo et al. describe four phenotypes of septic coagulopathy: cluster dA, with severe coagulopathy with extremely high levels of fibrinogen degradation products (FDP) and D-dimer, severe organ dysfunction, and the highest mortality rate, and particularly low platelet counts and prolonged prothrombin time (PT-INR); cluster dB, consisting in patients with severe disease, but moderate coagulopathy, intermediate levels of FDP and D-dimer and significant organ dysfunction; cluster dC, with moderate disease severity and moderate coagulopathy, and therefore relatively better clinical outcomes compared to the previous clusters; and finally, cluster dD, with mild disease with little to no coagulopathy, exhibiting the lowest levels of coagulation markers and the best prognosis among the groups [7].

The study explores the efficacy of recombinant human thrombomodulin (rhTM) as a potential treatment for septic coagulopathy. In Cluster dA, treatment with rhTM was associated with significantly lower 28-day mortality and in-hospital mortality [7]. However, these benefits were not observed in other phenotypes, highlighting the importance of phenotypic stratification in therapeutic decision-making. Other potential treatments for SIC include the use of anticoagulant therapies such as antithrombin supplementation and activated protein C (APC). However, clinical trials have produced mixed results, with some studies showing no significant reduction in mortality and others indicating an increased risk of bleeding [63,64]. The use of anticoagulants remains controversial, and current guidelines do not recommend routine antithrombotic therapy for all septic patients [62]. Instead, targeted therapies based on specific coagulation phenotypes may offer a more effective approach.

## 6. Conclusions

The phenotypic-based approach in sepsis management offers several advantages, opening avenues for future perspectives in personalized medicine. 

One of the primary advantages of a phenotypical approach is the ability to tailor interventions based on the specific characteristics of each phenotype, thereby enhancing the precision and effectiveness of treatments highlighting the pivotal role of artificial intelligence-based algorithms. 

However, the phenotypical approach is not without its limitations. One significant challenge is the inherent heterogeneity of sepsis, which complicates the identification and classification of distinct phenotypes. The vast variability in clinical presentations and biological responses necessitates sophisticated analytical tools and large datasets, which may not always be readily available in all clinical settings, making its usage difficult in the absence of a solid validation. Since the study of sepsis and septic shock is a continuously evolving field, the development of local guidelines based on the main four clinical features, such as patient-specific causes, trigger-related causes, clinical presentation, and response to therapy could be useful in clustering various septic patients for a better outcome. Thus, considering this clinically relevant variability, personalized treatment strategies could be more easily promoted.

Looking forward, the integration of artificial intelligence enables the analysis of larger datasets, playing a pivotal role in identifying novel clinical phenotypes. 

In conclusion, while the phenotypical approach in sepsis presents several advantages in terms of personalized patient care and improved prognostication, it also faces challenges related to heterogeneity and implementation in clinical practice. The future of sepsis management lies in the continued development and validation of phenotypical classifications, supported by advanced technologies and comprehensive data analysis, to ultimately achieve the goal of personalized medicine and improved patient outcomes.

## Figures and Tables

**Table 1:** Main studies focusing the patients’ comorbidities as specific causes of heterogeneity [4,5,6].

Study	Phenotypical Division	Conclusions
Seymour et al., 2019 [4]	α—Least organ dysfunction	Highest mortality in the δ phenotype (32%) Least mortality in the α phenotype (2%)
	β—Renal Dysfunction	
	γ—Hyperinflammatory status	
	δ—Liver dysfunction and coagulopathy	
Ding et al., 2021 [5]	Subgroup 1—Cardiovascular and respiratory dysfunction	Subgroup 2 had the highest mortality (28%), followed by Subgroup 1 (17%) and Subgroup 3 (10%)
	Subgroup 2—Renal, hepatic and respiratory dysfunction and increased inflammation - High in coagulopathy, deficiency anemias, liver disease and pulmonary circulation disease	
	Subgroup 3—Neurological dysfunction	
Taylor et al., 2022 [6]	(1) Low-risk, barriers to care	Highest mortality in the group 5 (8%) and the lowest mortality in group 1 (0.1%).
	(2) Previously healthy with severe illness and complex needs after discharge, barriers to care	
	(3) Multimorbidity - Elderly with malignancies and immunosuppression - Highest incidence of liver disease	
	(4) Poor functional status	
	(5) Existing poor health with severe illness and complex needs after discharge	

**Table 2:** Patient-specific causes of heterogeneity—age, temperature and immune response [8,10,11,12,13].

Study	Phenotypical Division			Conclusions
Shimazui et al., 2020 [10]	Elderly (>75 years old)			Non-elderly patients with a body temperature < 36.0 °C had significantly increased 90-day mortality.
	Non-Elderly (<75 years old)			
Ito et al., 2022 [11]	BMI	Low (<18.5 kg/m^2^)	Correlated with two groups of body temperature (BT): <36 °C and >36 °C	Highest mortality in normal BMI + hypothermia.
		Normal (18.5–24.9 kg/m^2^)		
		High (>24.9 kg/m^2^)		
Bhavani et al., 2019 [12]	Hyperthermic - Younger patients - Highest erythrocyte sedimentation rate (ESR) and C-reactive protein (CRP) levels.			Highest mortality in the hypothermic group. The hyperthermic, fast-resolvers group was associated with decreased mortality risk compared with the “normothermic group.
	Hyperthermic, fast resolvers			
	Normothermic			
	Hypothermic			
Baek et al., 2022 [13]	Cluster A—older age and lower BT			Mortality rate of Cluster A was significantly higher than that of Clusters B and C. Hypothermia was related to increased mortality regardless of age but significantly higher in the elderly
	Cluster B—younger age and wide range of BT			
	Cluster C—higher BT than Cluster A			
De Merle et al., 2024 [8]	Phenotype 1 - increased bilirubin, IL-6, ICAM - decreased white blood cell count and platelets			Phenotype 1 had greater 60-day inpatient mortality (41%)
	Phenotype 2 - Increased white blood cell count			

**Table 3:** Main studies focus on patients’ phenotyping with regard to the development of multi-organ dysfunction [4,5,29,30,31,32,33].

Study	Phenotypical Division	Conclusions
Knox et al., 2015 [29]	1. Shock with elevated creatinine	Highest mortality rates in shock with hypoxemia and altered mental status. Lowest mortality in shock with elevated creatinine.
	2. Minimal multi-organ dysfunction syndrome (MODS)	
	3. Shock with hypoxemia and altered mental status	
	4. Hepatic disease—marked coagulopathy	
Ibrahim et al., 2020 [30]	1. Liver disease	Highest mortality in cardiogenic and renal dysfunction, followed by cardiogenic dysfunction with hypoxemia and altered status.
	2. Cardiogenic and renal dysfunction	
	3. Minimal organ dysfunction	
	4. Cardiogenic dysfunction with hypoxemia and altered mental status	
Zhang et al., 2018 [31]	Profile 1—baseline type	Profile 3 had the highest mortality rate (45.4%), followed by profile 4 (27.4%), 2 (18.2%), and 1 (16.9%).
	Profile 2—respiratory dysfunction	
	Profile 3—multiple organ dysfunction (kidney, coagulation, liver, and shock)	
	Profile 4—neurological dysfunction.	
Seymour et al., 2019 [4]	α—Least organ dysfunction	Highest mortality in the δ phenotype (32%) Lowest mortality in the α phenotype (2%).
	β—Renal Dysfunction	
	γ—Hyperinflammatory status	
	δ—Liver dysfunction and coagulopathy	
Xu et al., 2022 [32]	1. Rapidly Worsening	Highest mortality in the Rapidly Worsening group was 28.3%.
	2. Delayed Worsening	
	3. Rapidly Improving	
	4. Delayed Improving	
Ding et al., 2021 [5]	Subgroup 1—Cardiovascular and respiratory dysfunction	Subgroup 2 had the highest mortality (28%), followed by Subgroup 1 (17%) and Subgroup 3 (10%).
	Subgroup 2—Renal, hepatic and respiratory dysfunction and increased inflammation	
	Subgroup 3—Neurological dysfunction	
Aldewereld et al., 2022 [33]	L1—Fluid refractory shock without multi-organ dysfunction	Highest mortality in the H2 group. $High incidence of intra-abdominal infections and bacteremia in H2 cluster.High requirements of vasopressors and mechanical ventilation in the H1 group.
	L2—Fluid responsive shock	
	M—Respiratory Failure	
	H1—Multiple organ dysfunctions, including cardiac and respiratory	
	H2—Liver Dysfunction and coagulopathy	

**Table 4:** Hemodynamic phenotypes in sepsis [12,38,41,43,44].

Study	Phenotypical Division	Conclusions
Geri et al., 2019 [38]	Cluster 1—Relative hemodynamic stability	ICU mortality was the highest in cluster 2.
	Cluster 2—LV systolic dysfunction	
	Cluster 3—Hyperkinetic profile with increased cardiac output	
	Cluster 4—RV failure	
	Cluster 5—Persistent hypovolemia	
Zhu et al., 2023 [41]	Class 1—SBP steady—about 100 mmHg	Patients in class 2 had the lowest SBP and the highest mortality risk (40.5%), while the lowest recorded mortality was in class 3, at 11.8%.
	Class 2—Stable SBP change trend, mean value 82 mmHg	
	Class 3—SBP gradually increased from 140 mmHg	
	Class 4—SBP steadily increasing from 110–120 mmHg to 120–130 mmHg	
	Class 5—SBP rapidly decreasing from 130 mmHg to 100 mmHg	
	Class 6—SBP rapidly decreasing from 150–160 mmHg to 110–120 mmHg	
	Class 7—SBP initially on an increasing trend, followed by decreasing. Average SBP > 160 mmHg	
Shald et al., 2021 [43]	Multi-organ Failure (MOF)	MOF and ND had the highest volume balance and mortalities, at 48.4% for MOF and 39.7% for ND, followed by RD (20.8%) and OP (13.7%).
	Respiratory Dysfunction (RD)	
	Neurological Dysfunction (ND)	
	Other patients (OP)	
Ma et al., 2021 [44]	1. Baseline - early norepinephrine	Highest mortality in cluster 2 (40%), lowest in cluster 5 (21%).
	2. Critical - fluid resuscitation and delayed norepinephrine	
	3. Renal dysfunction - early norepinephrine and fluid de-resuscitation by day 1	
	4. Respiratory failure - restrictive fluid management	
	5. Mild	
Bhavani et al., 2022 [12]	Group A—hyperthermic, tachycardic, tachypneic, hypotensive	Highest mortalities in groups A and D.
	Group B—hyperthermic, tachycardic, tachypneic, hypertensive	
	Group C—hypothermic, tachycardic, tachypneic, normotensive	
	Group D—hypothermic, tachycardic, tachypneic, hypotensive	

## Data Availability

No new data were created or analyzed in this study. Data sharing is not applicable to this article.
